# Novel Culturing Techniques Select for Heterotrophs and Hydrocarbon Degraders in a Subantarctic Soil

**DOI:** 10.1038/srep36724

**Published:** 2016-11-09

**Authors:** J. M. van Dorst, G. Hince, I. Snape, B. C. Ferrari

**Affiliations:** 1 School of Biotechnology and Biomolecular Sciences, UNSW Australia, 2052, NSW, Australia; 2 Australian Antarctic Division, Department of the Environment, 205 Channel Highway, Kingston, TAS 7050, Australia

## Abstract

The soil substrate membrane system (SSMS) is a novel micro-culturing technique targeted at terrestrial soil systems. We applied the SSMS to pristine and diesel fuel spiked polar soils, along with traditional solid media culturing and culture independent 454 tag pyrosequencing to elucidate the effects of diesel fuel on the soil community. The SSMS enriched for up to 76% of the total soil diversity within high diesel fuel concentration soils, in contrast to only 26% of the total diversity for the control soils. The majority of organisms originally recovered with the SSMS were lost in the transfer to solid media, with all 300 isolates belonging to *Proteobacteria*, *Firmicutes*, *Actinobacteria* or *Bacteroidetes*, the four phyla most frequently associated with soil culturing efforts. The soils spiked with high diesel fuel concentrations exhibited reduced species richness, diversity and a selection towards heterotrophs and hydrocarbon degraders in comparison to the control soils. Based on these observations and the unusually high level of overlap in microbial taxa observed between methods, we suggest the SSMS holds potential to exploit hydrocarbon degraders and other targets within simplified bacterial systems, yet is inadequate for soil ecology and ecotoxicology studies where identifying rare oligotrophic species is paramount.

Microbial populations have been proposed as valuable indicators of polar soil health^1^,^2^,^3^. They constitute the bulk of the biomass in polar soils and as such, are primarily responsible for ecosystem services such as decomposition, mineralisation, inorganic nutrient turnover and pollutant removal^4^. Rapid sequencing through the use of next generation sequencing (NGS) platforms have evaluated the impact of petroleum hydrocarbons on bacterial community diversity in mangroves^5^, oil fields^6^, the Gulf of Mexico following the Deepwater Horizon oil spill^7^ and contaminated soils in the sub-Antarctic^8^. It has been consistently demonstrated that the presence of petroleum hydrocarbons shift the phylogenetic make up of communities^9^,^10^,^11^. However, understanding the functional relevance of these phylogenetic shifts remains problematic. Cultured representatives provide greater insight into individual and community microbial functions and have the potential to validate sequence-based observations through functional experimentation.

The uncultured majority are thought to have oligotrophic growth strategies^12^ and grow predominantly as micro-colonies^13^,^14^,^15^. By mimicking the oligotrophic conditions found in a specific environment, novel micro-culturing techniques have begun to bridge the gap between the cultured, and uncultured majority^16^,^17^,^18^,^19^,^20^,^21^. Novel culturing strategies are aimed at limiting nutrient availability, increasing incubation times and providing direct contact with the source environment^13^,^14^,^20^. Approaches designed to mimic aquatic environments have resulted in the successful growth of bacteria from many divisions, including the candidate divisions SAR11^17^ and OP10^13^,^16^. The soil substrate membrane system (SSMS) is a novel micro-culturing technique targeted at terrestrial systems^22^. The technique mimics *in-situ* growth conditions of the soil environment by using environmental soil as the growth substrate, replacing the excessive nutrients characteristic of artificial media. The SSMS has been demonstrated to substantially increase the culturability of soil bacteria with reports of up to 40% of culture-independent diversity captured with the membrane enrichments^21^,^22^,^23^.

Macquarie Island is a remote Australian sub-Antarctic territory located in the Southern Ocean (54°37′53″S, 158°52′15″E) (Fig. 1). After a tumultuous history of exploitation from sealers, the island was declared a wildlife sanctuary in 1933 and has been operating as permanent Australian Antarctic research station since 1948. The continual human occupation has led to the presence of contaminants in the air, sea and soil, with petroleum hydrocarbons identified as one of the most prolific^24^. Three major petroleum hydrocarbon contaminated sites have been identified on Macquarie Island surrounding the fuel storage and power generation facilities (Fig. 1)^25^,^26^. A bioremediation program began in 2009 to remediate the historical and recent fuel spills, utilising nutrient addition and bioventing to stimulate the petroleum hydrocarbon degradation potential of the indigenous microbial population^25^,^27^,^28^.

In order to inform bioremediation, ecotoxicology, and microbial ecology investigations, our aim was to utilise the SSMS to increase the culturability of the bacterial communities present in sub-Antarctic soils. We compared the results from SSMS culturing against direct culturing with artificial media, and both culturing techniques against a culture independent method; pyrosequencing targeting the *16S rRNA* gene. Furthermore, by spiking the soil across a range of diesel fuel concentrations, we evaluated how the presence of diesel fuel altered the sub-Antarctic soil bacterial community.

## Methods

### Site and sampling methods

**Figure 1:**
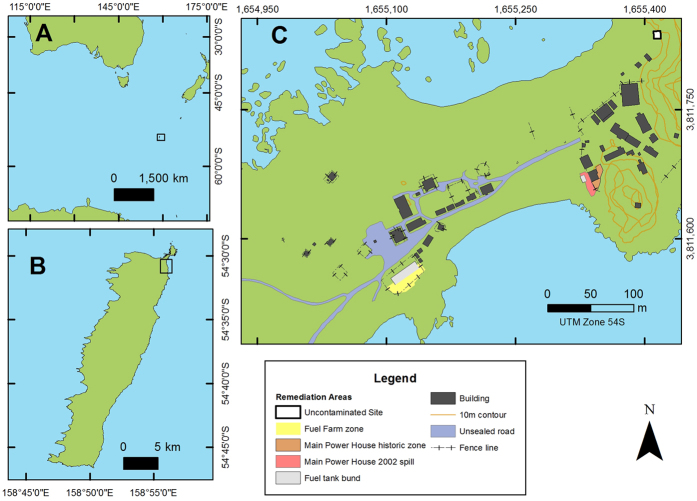
Location of (**A**) Macquarie Island in the Southern Ocean, (**B**) the ANARE research station at the Northern tip of the island and (**C**) the research station layout. The yellow, red and orange sites on the map indicate the contaminated sites currently undergoing remediation. The uncontaminated sampling site is indicated by the white rectangle outlined in black. Station buildings are shown by black rectangles. This map was created in ArcGIS Version 10.3 https://esriaustralia.com.au/products-arcgis-software-10-3.

At Macquarie Island, a soil sample (500 g) was collected from the top 30 cm of an uncontaminated plot (−54.4974°, 158.9395°) approximately 170 m away from the largest contaminated site at the Main Power House (MPH) (Fig. 1)^25^. The soil was sub-sampled and spiked with Special Antarctic Blend (SAB) diesel fuel over the range of 0–20 000 mg kg^−1^ (Table 1). The soil samples were homogenised and stored for 3 months at ambient temperature (10 °C). To determine total petroleum hydrocarbon (TPH) concentrations after spiking, sub-samples (10 g) from each spiked soil were extracted with hexane and assessed by gas chromatography flame ionisation detection (GC-FID), according to the method described in Schafer *et al.*^29^. As organisms usually respond logarithmically to toxicants, the spiked soils were grouped into the following biologically relevant diesel fuel concentration ranges; control (no fuel additions), low (DL-400 mg kg^−1^), medium (400–5000 mg kg^−1^), and high (>5000 mg kg^−1^) (Table 1). An adjacent core was collected to determine the nutrient and chemistry parameters of the soil according to the methods described in Mooney *et al.*^30^. After the 3 month incubation period, soil samples were sieved aseptically through a 2 mm metal sieve to remove large particulate matter in preparation for cultivation and micro-cultivation.

### Traditional cultivation on artificial media

To create the bacterial inoculums, a sub-sample of each sieved soil was diluted 1:200 in filtered distilled H_2_O, vortexed for 30 s and allowed to settle. Serial dilutions of the inoculum were created with filtered distilled H_2_O at 10^−1^, 10^−2^ and 10^−3^. An aliquot (100 μl) of each serial dilution was spread onto low nutrient artificial media (0.01 × RAVAN) plates in triplicate according to Ferrari *et al.*^22^. Plates were sealed with parafilm to prevent moisture loss and then incubated aerobically and anaerobically at 10 °C. For anaerobic conditions the plates were placed in an anaerobic chamber (Becton Dickinson, Australia) with anaerobic gas packs (BD). The resulting macro-colonies were sub-cultured (at least 3X) until pure isolates were obtained, paying particular attention to unique morphologies.

### Enrichments with the SSMS

For micro-cultivation, 50 μl of the 1:200 inoculums from above, was added to 10 ml of distilled H_2_O, then filtered onto a 0.2 μm, white, Isopore PC membrane (Millipore, Australia) with a vacuum pump filtration manifold as described in Ferrari *et al.*^31^. The top of the sterile TCI membrane served as a barrier between the non-sterile soil slurry and the underside of the sterile PC membrane.

For each individually spiked soil sample (Table 1), six replicate SSMS were set up in 6 well plates: three for aerobic and three for anaerobic incubation conditions and incubated at 10 °C. The optimal incubation time of 16 days was determined by evaluating growth membranes for micro-colonies, without overcrowding, as determined through epi-fluorescent microscopy (EFM)^22^. For aerobic conditions, the final culture vessels (6 well plates) were hydrated and sealed to prevent moisture loss during incubation^22^. For anaerobic conditions, the vessels were hydrated and placed in an anaerobic chamber with anaerobic gas packs. After 16 days of incubation, the membranes were removed and microbial growth was confirmed with EFM. After growth confirmation, the remaining membrane quarters were combined for each sample and used for DNA extraction and secondary culturing on artificial media.

### Secondary cultivation from the SSMS enrichments onto artificial media

Following micro-cultivation, the tips of the remaining quarters of growth membrane were secured in the lid of 1.5 ml tubes and vortexed with 1 ml of filtered Tris-EDTA buffer to remove the cells from the membrane. The buffer was centrifuged for 60 s at 13 000 g to create a pellet and the supernatant was removed. The pellet was resuspended in 300 μl TE buffer and 100 μl was used as an inoculum to spread in triplicate on low nutrient media plates (0.1 × RAVAN). This process was repeated for all the samples. The resulting macro-colonies were sub-cultured until pure cultures were obtained.

### DNA extraction and RFLP of pure isolates

DNA was extracted from pure isolates by boiling cells at 100 °C for 10 min, followed by centrifugation at 13,000 g for 15 min. To amplify the *16S rRNA* gene the resulting lysate was transferred to a clean tube for PCR and 10 ng was added to 50 μl PCR reactions using primers F27 and R1492^32^. The PCR protocol consisted of initial denaturation at 94 °C for 5 min, followed by 35 cycles of 94 °C for 30 sec, 60 °C for 30 sec and 72 °C for 30 and terminated with a final step of 72 °C for 5 min. A portion (15 μl) of the *16S rRNA* PCR amplicons was digested with the restriction enzymes *HinFl* and *Rsal* (Promega, USA) following the manufacturer’s instructions. Digested products were visualised on a 2% agarose gel after staining with SYBR safe. Unique RFLPs were selected for *16S rRNA* gene sequencing. For RFLPs observed >5 times, PCR products were sourced from more than one isolate.

### Sanger sequencing of pure cultured isolates

The PCR products of selected isolates were purified using a PCR purification kit (Qiagen, USA). Approximately 30 ng of the purified PCR product was utilised in a sequencing reaction with 1 μl BigDye Terminator (Applied Biosystems) and 1.5 μl 5X buffer, per 20 μl reaction. The sequencing reaction protocol consisted of 25 cycles of 96 °C for 10 sec, 50 °C for 5 sec and 60 °C for 4 min. Reaction products were cleaned with ethanol and EDTA (Ethylene diamine tetra-acetic acid) and analysed with an ABI 3730 sequence scanner at the Ramaciotti Centre for Gene Function Analysis (UNSW Australia). All sequence data from the ABI sequencer was checked for chimeric artefacts using the program bellapheron http://foo.maths.uq.edu.au/~huber/doc/doc/bellerophon.pdf. The data was then analysed using the NCBI nucleotide Basic Local Alignment Search Tool (BLAST) for nearest matches and closest cultured isolates in the Genbank Database.

### DNA extraction from SSMS enrichments

The mixed bacterial community present on the enriched SSMS growth (PC) membranes were used for DNA extraction. The remaining membrane quarters were added to a 2 ml lysing matrix tube from the FastDNA SPIN kit for soil (MP Biomedicals, USA) and extracted according to the manufacturer’s instructions. To confirm the presence of DNA in the extracts a portion of each sample (6 μl) was electrophoresed on a 2% agarose gel. The DNA extracted from individual growth membranes was combined to generate one aerobic and one anaerobic sample for pyrosequencing at each concentration, for a total of 14 samples (Table 1).

### DNA extraction from soil

After preliminary optimisation on the extraction of gDNA from polar soils, the FastDNA SPIN kit for soil (MP Biomedicals, Seven Hills, NSW, Australia) was selected over other commercial soil DNA extraction kits based on higher DNA yields, and the amount of soil used for the extractions was reduced from 0.5 g to approximately 0.25–0.30 g (data not shown). Each soil sample was extracted in triplicate and gDNA was quantified using a Picogreen assay (Life Technologies, Australia), with absorbance measured using a fluorescence plate reader (SpectraMax M3 Multi-Mode Microplate Reader, Molecular Devices, USA). In order to account for extraction, amplification and sequencing variability within samples, all three independently extracted DNA replicates were sequenced for the un-spiked control soil (Table 1).

### Pyrosequencing targeting the 16S rRNA gene

The extracted DNA were purified and amplified for 454 FLX titanium pyrosequencing with the *16S rRNA* gene universal primers; *28F* and *519R* according to the methods outlined in Dowd *et al.*^33^. Raw sequence data was provided in the form of standard flowgram format (sff) files. The sequences and flowgrams were extracted from the sff files, de-multiplexed and error-checked via the Pyronoise algorithm^34^ in MOTHUR^35^. Further quality screening of the sequence data was completed according to the pipeline in van Dorst *et al.* Bacterial seed sequences were aligned to the curated SILVA secondary structure alignment^36^. Aligned 16S sequences were then clustered into OTUs based on 96% sequence similarity as the best definition for species-level OTUs in this region of the 16S gene^37^. Taxonomic assignment of the identified bacterial OTUs was performed using the Greengenes 2011 database trimmed to the same region as our amplicons (V1-V3)^38^. An OTU abundance-by-sample matrix was generated from the bacterial dataset with MOTHUR^35^. As an additional stage of quality control, global *16S rRNA* gene sequence singletons were removed from the dataset and sequences were subsampled to the lowest number of reads pre sample (1950). The final processed sequences were deposited into NCBI database under the project accession number PRJNA338201.

### Calculation of diversity estimates from pyrosequencing data

To evaluate the overall bacterial diversity recovered from the SSMS following pyrosequencing samples, aerobic and anaerobic sequences were combined for each fuel concentration analysed. Multivariate data analysis was conducted with the software packages PRIMER v6 and Permanova+. To account for the log-normal distribution of the data, the abundance-by-sample matrix was square root transformed. After transformation the skewness and kurtosis was reduced closer to 0. For sample comparison the abundance-by-sample matrix was then standardised to express the OTU abundances as relative abundances. The community diversity estimates of richness, Pielou’s evenness coefficient (J), Shannon (H’) and Simpson (λ) were calculated in PRIMER. To interrogate the shared diversity between the SSMS enrichments and the soil samples, the consistent OTU’s were identified and multiplied by their relative abundance. The resulting shared diversity was calculated for each diesel fuel concentration range and expressed as a percentage of the total soil diversity. A two-tailed student’s T-test was used to test the null hypothesis that diesel fuel concentration made no difference in the shared diversity between methods.

### Response of individual genera to diesel fuel

The log abundance of individual genera was plotted against the log TPH concentration. Linear equations were fitted to the observed dose response and solved to determine the slope of the line. The fit of the linear model was assessed by calculating a sum of squares test with significance set at *P* < 0.05. The results were summarised in a volcano plot where the x-axis indicated the slope of the line (negative values infer inhibition and positive values infer stimulation), and the y-axis indicated the −log (*P* value). Calculating the –log of the *P* values allowed the genera with the greatest linear relationship to TPH to be positioned towards the top of the plot, and individual genera with the most substantial shifts in relative abundances positioned furthest from the middle of each plot.

## Results

### Comparison of culture dependent verses independent methods to capture bacterial diversity

**Figure 2:**
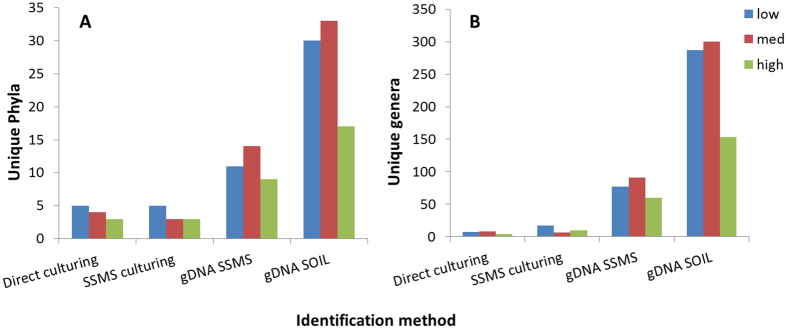
The number of unique phyla (**A**) and genera (**B**) recovered with culture dependent and culture independent techniques. Numbers recovered are grouped by diesel fuel concentration ranges; low (DL-400 mg kg^−1^), medium (401–5000 mg kg^−1^) and high (>5000 mg kg^−1^). The total extraction of gDNA from SSMS enrichments and directly from soil retrieved considerably more species than the culturing techniques. The pyrosequencing results revealed a peak in unique phyla and genera at mid-level diesel fuel concentrations.

As expected, molecular approaches sequencing total gDNA (directly from the soils or SSMS enrichments) recovered far greater numbers of species, genera and phyla than the pure culture dependent methods alone (Fig. 2, Table 2). Of the pure culture dependent approaches, the SSMS and artificial media combination recovered more genera and species than culturing directly from the soil alone. Yet both cultivation approaches recovered members from the same four phyla; *Proteobacteria*, *Firmicutes*, *Actinobacteria* and *Bacteroidetes*.

### Diversity of pure cultured isolates

A total of 300 isolates were recovered from the soil using artificial media and from the combination of the SSMS and artificial media. From the 300 isolates, 69 RFLPs were identified as unique and following sequencing and a total of 46 species from 24 genera were identified (Table 3). The SSMS and artificial media combination recovered a greater number of species in comparison to the traditional culturing directly from soil, with 32 species from 18 genera isolated compared to 18 species and 11 genera respectively. Of the 69 unique RFLPs identified, 10 were unable to be successfully sequenced. The majority of isolates recovered belonged to the genera *Arthrobacter*, *Sphingomonas* and *Pseudomonas* with 89, 60 and 43 isolates respectively. There were 5 novel isolates unable to be accurately identified below genus level with the following closest matches; *Achromobacter xylosoxidans 96*%, *Arthrobacter psychrochitiniphilus 96*%, *Arthrobacter stackebrandtii 96*% *Hymenobacter ocellatus 96*% *Sphingomonas faeni 95*% and *Yersinia enterocolitica 96*%, and 2 novel isolates were unable to be identified below family level with the following closest matches; *Adhaeribacter terreus 91*%, *Arthrobacter sulfureus 94*%.

Of the pure cultured genera, 4 were more dominant in the low TPH contamination samples including *Acidovorax*, *Brevundimonas*, *Microbacterium* and *Sporasarcina*, while the genera *Rhodococcus* were particularly prevalent in the mid-range TPH samples (Table 3). The genera *Arthrobacter*, *Burkholderia*, and *Sphingomonas* were dominant across all TPH ranges. *Pseudomonas* and *Rhodanobacter* were also present in all TPH ranges but increased their prevalence in the samples with high TPH, along with *Achromobacter*. Of the genera recovered more than once, *Microbacterium*, *Paenibacillus* and *Burkholderia* were recovered only with the SSMS to artificial media combination, and *Achromobacter* was recovered only with the direct culturing from soil.

### Pyrosequencing diversity from the SSMS enrichments and soil samples

For the SSMS enrichments a total of 34,308 *16S rRNA* gene sequences across the 14 samples (Table 1) were obtained. After filtering out the low quality sequences a total of 24,494 (average 1,749 per sample) remained. The results from the aerobic and anaerobic samples at each concentration were combined, resulting in 7 samples with a new average of 3,498 sequences per sample. For the soil samples, a total of 45, 322 *16S rRNA* gene sequences across all 9 soil samples (Table 1) were obtained. After filtering out the low quality sequences a total of 32,358 sequences (average 3,595 per sample) remained. For further community diversity analysis, all samples were subsampled to 1950 sequence reads.

**Figure 3:**
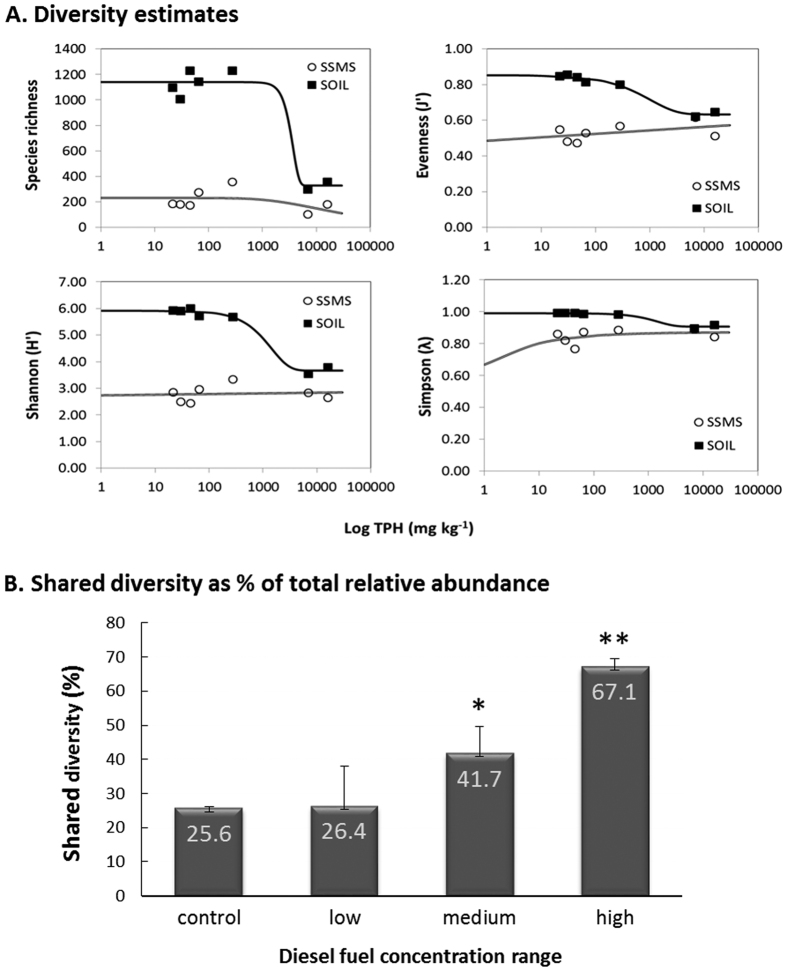
Community diversity estimates (**A**) and shared taxonomic diversity (**B**) against the diesel fuel concentration gradient. (**A**) The SSMS enrichments exhibited 17.9% of the total species richness recovered directly from the soil. For the soil samples a decline in species richness, evenness, and Shannon and Simpson diversity indices was observed with increasing TPH. At the highest TPH concentrations, all indices were similar between the soil and the SSMS enrichments. (**B**) The shared taxonomic diversity was found to be significantly greater in the medium *(*P* < 0.05) and high **(*P* < 0.005) diesel fuel concentration ranges when compared to the control.

As expected the species richness was higher in the soil community in comparison to the enriched SSMS community (Fig. 3A). On average, the species richness in the control SSMS enrichments was 17.9% of the richness found in the control soil samples, which increased to an average of 42.16% for the high diesel fuel concentration range. The species evenness (J’), Shannon (H’) and Simpson (λ) diversity estimates were also greater in the soil communities. Within the soil samples, all community estimates measured declined with increasing TPH. In contrast, the response within the SSMS enrichments was negligible, with small increases in the evenness and Simpson estimates and a decline in the species richness. Expressed as a percentage of the total soil diversity, the shared diversity ranged from an average of 25.6–67.1% (Fig. 3B). The percentage of shared OTUs was found to significantly increase from the control soils to the medium and high diesel fuel concentration ranges. Overall, the samples within the high TPH concentration range had the highest percentage of shared diversity at the OTU level (67.1%) and the greatest similarity across all measured diversity estimates (Fig. 3A).

### Bacterial phylogenetic diversity from the SSMS enrichments and soil samples

**Figure 4:**
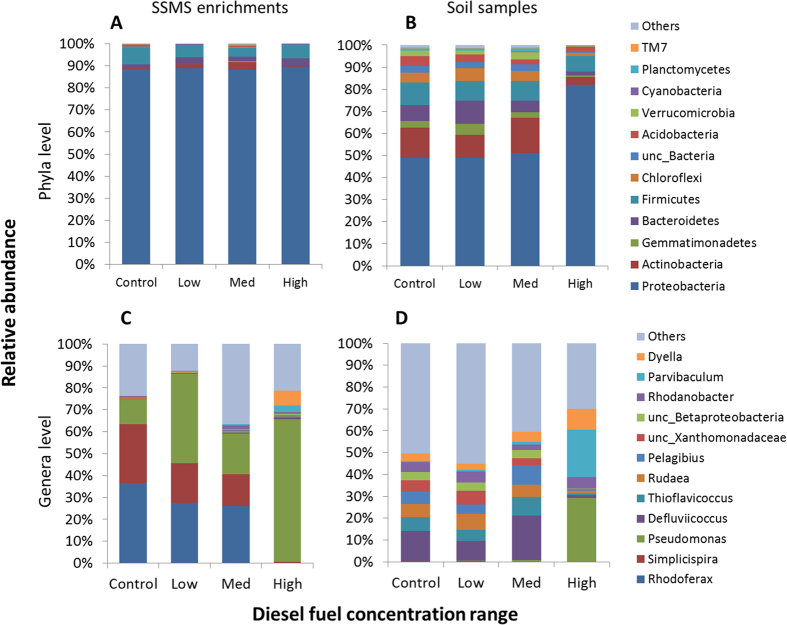
Relative abundances for SSMS enrichments and soil samples across diesel fuel concentration ranges at the Phyla (**A**,**B**) and Genera (**C**,**D**) level diversity. For both approaches *Proteobacteria* was the dominant phyla and *Pseudomonas* was the dominant genus, particularly within high TPH concentration samples.

At the phyla level the SSMS enrichments were dominated by *Proteobacteria*, contributing 88–91% of the total relative abundance, followed by *Firmicutes* (2–10% of the total relative abundance), and *Actinobacteria*, *Bacteroidetes*, and *Acidobacteria* (<5% relative abundance) (Fig. 4A). The total soil diversity was also dominated by species within *Proteobacteria*. However, the detected prevalence was lower than that estimated for the SSMS enrichments, ranging between 49–51% of the total relative abundance for the control, low and medium diesel fuel concentrations (Fig. 4B). Other phyla that were abundant in the control, low and medium diesel fuel range soils included *Firmicutes* (7–11%), *Actinobacteria* (9–18%), *Bacteroidetes* (5–12%), *Gemmatimonadetes* (2–3%), *Acidobacteria* (2–5%), and *Chloroflexi* (4–6%). The phyla *Planctomycetes*, *Verrucomicrobia*, *Cyanobacteria*, Candidate Division *TM7* and the remaining phyla denoted ‘Others’ were also present, contributing less than 5% each of the total relative abundance. In the high diesel fuel ranges, the composition for the soil samples changed dramatically with *Proteobacteria* contributing to over 80% of the total relative abundance. Apart from *Firmicutes* at 8% of the relative abundance in the highest fuel concentration, all remaining phyla contributed <5% each to the total relative abundance observed.

Within *Proteobacteria*, the control, low and medium diesel fuel concentration ranges for the SSMS enrichments were dominated by *Rhodoferax* (26–35%) and *Simplicispira* (12–25%), yet within the high diesel fuel range both genera were barely detected (Fig. 4C). *Pseudomonas* was prevalent across all fuel concentrations but dominated the high-range diesel fuel samples contributing over 60% of the total relative abundance observed. For the high diesel fuel range *Dyella* (3–10%), *Parvibaculum* (1–4%) and *Rhodanobacter* (1%) were also detected (Fig. 4C). Within the soil samples, the community composition of the control, low and medium diesel fuel concentration ranges was found to be vastly different to the equivalent SSMS enrichments at the genera level diversity (Fig. 4D). The genera *Simplicispira* and *Rhodoferax* were barely detected and instead *Delfuviicoccus* was the most abundant genera contributing between 8–20% of the total relative abundance. A greater number of genera were detected in relatively low abundances in soil; *Thioflavicoccus* (3–8%), *Rudaea* (2–4%), *Pelagibius* (3–10%), unclassified species within *Xanthomonadaceae* (2–5%), unclassified species within *β-Proteobacteria* (3%), *Rhodanobacter* (1–3%) and *Dyella* (1–4%). Additionally, a further 305 genera represented by ‘Others’ contributed to a combined level of 40–55% of the total relative abundance. In the highest diesel fuel concentration range, there was a substantial decline in the number of total detected genera within the soil samples (Fig. 4D). Although present in different proportions, the major genera recovered within the high TPH concentration range were consistent between the SSMS enrichments and soil samples, in particular *Pseudomonas*, *Rhodanobacter*, *Parvibaculum* and *Dyella*.

### Response of individual genera to TPH

**Figure 5:**
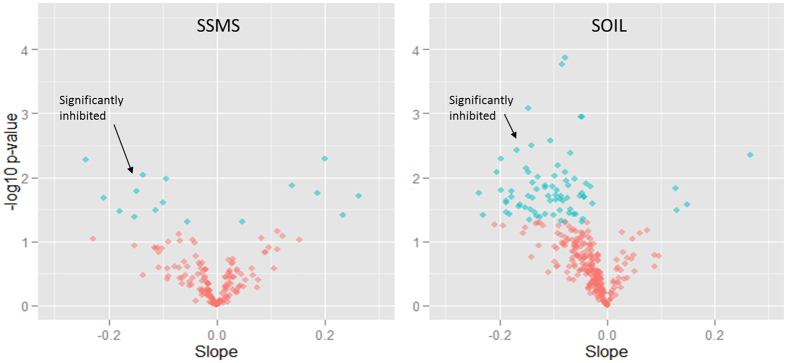
A volcano plot of the genera significantly inhibited or stimulated with increasing diesel fuel concentrations. Blue diamonds indicate a significant response at p < 0.05, pink diamonds were non-significant. On the x-axis, negative slope values indicate inhibition and positive values indicate stimulation. A greater number of inhibited genera were detected with the culture independent method.

Analysis of the response of bacterial genera present in the SSMS enrichments to TPH revealed 12 genera were significantly inhibited and 6 genera were significantly stimulated (*p* < 0.05) (Fig. 5, Table 4). In contrast, the genera identified in soil had 71 genera significantly inhibited and only 5 genera significantly stimulated. All of the genera significantly stimulated with increasing diesel fuel concentrations were members of *Proteobacteria* (Table 4), and the majority of genera were consistent between the two methods (*Pseudomonas*, *Parvibaculum*, *Herbaspirillum* and *Dyella*). However, the genera significantly inhibited with TPH originated from a total of 6 different phyla, and despite the larger numbers, there were no organisms identified as significantly inhibited within both the soil and SSMS enrichments (Table 4).

## Discussion

The SSMS enrichments combined with secondary cultivation onto solid media recovered greater numbers of genera and species, and a greater number of novel isolates than artificial media alone. However, neither of the cultivation–dependent methods recovered isolates belonging to phyla outside *Proteobacteria*, *Firmicutes*, *Actinobacteria* or *Bacteroidetes* (Table 2). Organisms belonging to these four phyla are the most commonly cultured species from the environment, including extreme polar environments^39^. However, culturing soil bacteria from phyla outside of these four well-characterised phyla is possible, and has been achieved through the use of dilute media and extended incubation times^40^. Novel cultivation methods that mimic the natural environment continue to reduce the gap between culture-dependent and culture-independent approaches^21^,^23^,^41^, expanding our knowledge of novel species and novel compounds. For example, in Ling *et al.*, the successful domestication of a slow growing and previously uncultured bacteria led to the discovery of a novel antibiotic, Teixobactin^41^.

Here, the SSMS enrichments generated a considerable proportion (18% to 42%) of the total species richness, which is consistent with reported recoveries^21^. Yet, only a limited percentage (~7%) of the OTU’s were consistent with the control soils and neither of the two most dominant Genera (*Simplicispira* and *Rhodoferax*) were recovered. Additionally, only a small proportion of this diversity was successfully transferred to solid media, comparable to results obtained when using artificial media alone. In the future, further dilutions of our low nutrient SAB media, combined with dilution-to-extinction approaches may help improve the success rate of transferring micro-colonies from the SSMS enrichments to isolated macro-colonies. Furthermore, we suggest that combining the SSMS with single-cell applications such as flow cytometry^42^,^43^ and micro- manipulation^43^, is a more appropriate method of domesticating rare organisms from extreme environmental samples such as polar soils.

The addition of diesel fuel is known to reduce phylogenetic diversity and selects for hydrocarbon degraders and heterotrophs^11^. This trend was expected and observed across all diesel fuel concentration ranges and methods utilised. Within the high diesel fuel soils, both the cultivation dependent and independent methods recovered *Pseudomonas* and *Rhodanobacter*, while the SSMS and total soil communities also recovered *Dyella* and *Parvibaculum*^8^,^9^,^10^,^11^. The selective pressure from the addition of diesel fuel also reduced the total species richness, evenness and diversity within the soils, as calculated by Shannon, Simpson, and Pileu diversity indices (Fig. 3). Overall, the SSMS enrichments exhibited substantially lower species richness, evenness, and phylogenetic diversity than the corresponding soil samples. This was particularly true for the control and low diesel fuel concentration ranges. However, the reduction in community complexity, combined with the selection towards heterotrophs following diesel fuel additions, resulted in the SSMS enrichments more closely reflecting both the phylogenetic diversity and community structure of the high diesel fuel concentration samples (Fig. 3). The three most dominant genera (*Pseudomonas*, *Rhodanobacter* and *Dyella*) were consistent between both methods and made up and average of 60% and 70% of the total relative abundance in the SSMS enrichments and soil samples respectively. Overall, 67% of the total diversity was consistent between methods at the highest fuel concentrations, in contrast to just 26% for control soils. Based on these observations, we believe the SSMS may be a valuable tool to investigate the bacterial community, and to target hydrocarbon degraders or other species of interest within simplified heterotrophic communities^21^,^23^.

The majority of genera identified from the soil were present in very low abundances and were significantly inhibited (p < 0.05) with increasing diesel fuel concentrations (Fig. 5). This is consistent with previous observations^8^. There is evidence that vital soil ecosystem services are carried out by organisms present in low abundances and that these organisms may be particularly vulnerable to disturbances^8^,^44^. For example, when examining the ecophysiology of anaerobic phototrophic bacteria, Musat *et al.*, revealed that species representing approximately 0.3% of the total cell number contributed more than 40% of the total uptake of ammonium and 70% of the total uptake of carbon in the system. While in van Dorst *et al.*, species essential to nitrification present in sub-Antarctic soils at <1% relative abundances, were shown to be the most sensitive indicator of toxicity to diesel fuel. Thus, in order to capture the breadth of microbial ecosystem functions and the ecological implications of contamination and other disturbances, it is essential to be able to identify the depth and breadth of organisms present in the ecosystem in question.

Consistent with traditional culturing techniques^12^, the SSMS appears to maintain a selective bias towards heterotrophic species. While the bias towards heterotrophs remains, we suggest the SSMS is unsuitable to characterise total soil bacterial communities. As discussed earlier, the capacity to recover species in low abundance and species with oligotrophic growth strategies is crucial for ecotoxicology^4^,^8^,^45^,^46^ and soil ecology investigations^47^,^48^,^49^,^50^. To that end, extracting total gDNA directly from the soil and targeting taxonomic markers on one of the current sequencing platforms remains the ideal method to profile microbial communities. It is important to note that except in the case of functionally specific clades, phylogenetic diversity offers limited information on the functional capabilities and the ecological relevance of community shifts that occur in response to contamination events. At this stage, representative functional data remains limited (particularly in underrepresented environments) for the classification of microbial communities into biologically meaningful categories^51^. Alternatively, methods that target the functional potential or ecological significance of microbial dynamics such as qPCR^8^, network analysis^45^, and shotgun metagenomics are useful strategies to combine with pyrosequencing in order to further illuminate the ecological relevance of microbial interactions in soil.

## Additional Information

**How to cite this article**: van Dorst, J. M. *et al.* Novel Culturing Techniques Select for Heterotrophs and Hydrocarbon Degraders in a Subantarctic Soil. *Sci. Rep.* **6**, 36724; doi: 10.1038/srep36724 (2016).

**Publisher’s note**: Springer Nature remains neutral with regard to jurisdictional claims in published maps and institutional affiliations.

## Figures and Tables

**Table 1:** Nominal and measured diesel fuel concentrations, incubation conditions and numbers of samples sent for tag pyrosequencing.

Nominal TPH mg kg^−1^	Measured TPH mg kg ^−1^	Diesel fuel concentration range	SSMS Incubation conditions	Number of soils for SSMS/pyrosequencing
0	<D.L.	low	aerobic/anaerobic	2/3
100	96	low	aerobic/anaerobic	2/1
250	274	low	aerobic/anaerobic	2/1
500	588	medium	aerobic/anaerobic	2/1
1000	1256	medium	aerobic/anaerobic	2/1
10000	9398	high	aerobic/anaerobic	2/1
20000	16815	high	aerobic/anaerobic	2/1

**Table 2:** Diversity of bacterial members at Phylum level using culture dependent and culture independent methods.

Artificial media culturing	SSMS and artificial media culturing	gDNA from SSMS enrichments	gDNA from soil
*Proteobacteria*	*Proteobacteria*	*Proteobacteria*	*Proteobacteria*
*Firmicutes*	*Firmicutes*	*Firmicutes*	*Firmicutes*
*Actinobacteria*	*Actinobacteria*	*Actinobacteria*	*Actinobacteria*
*Bacteroidetes*	*Bacteroidetes*	*Bacteroidetes*	*Bacteroidetes*
		*Gemmatimonadetes*	*Gemmatimonadetes*
		*Acidobacteria*	*Acidobacteria*
		*Chlorobi*	*Chlorobi*
		*Chloroflexi*	*Chloroflexi*
		*Verrucomicrobia*	*Verrucomicrobia*
		*Spirochaetes*	*Spirochaetes*
		*Planctomycetes*	*Planctomycetes*
		*TM7*	17 × Candidate divisions
			18 × Others

**Table 3:** Pure cultured isolates recovered on artificial media from soil or the SSMS enrichments.

Isolate number	No. of RFLPS	Soil SSMS	TPH Conc. Range	Closest cultured representative	% Similarity
66	1	Soil	High	*Achromobacter xylosoxidans*	98
20A	(4)	Soil	High	*Achromobacter xylosoxidans*	99
20B	(4)	Soil	High	*Achromobacter xylosoxidans*	96
54	1	SSMS	Control	*Acidovorax radicis strain*	98
60	3	Artificial	Control	*Acidovorax wohlfahrtii*	99
61	1	Artificial	Med	*Adhaeribacter terreus*	91
36	2	Artificial	Med	*Arthrobacter psychrochitiniphilus*	96
3A	(3)	SSMS	Low	*Arthrobacter stackebrandtii*	96
3B	(3)	SSMS	High	*Arthrobacter psychrolactophilus*	99
3C	(3)	Artificial	Control	*Arthrobacter psychrolactophilus*	99
1A	(79)	SSMS	High	*Arthrobacter psychrolactophilus*	99
1B	(79)	SSMS	Low	*Arthrobacter stackebrandtii*	97
31	2	SSMS	Low	*Arthrobacter stackebrandtii*	99
33	1	SSMS	Low	*Arthrobacter stackebrandtii*	98
52	1	SSMS	Low	*Arthrobacter sulfureus*	94
55	1	SSMS	Control	*Bosea massiliensis*	98
25	1	SSMS	Low	*Brevundimonas bullata*	99
26	1	Artificial	Low	*Brevundimonas staleyi*	98
27	1	SSMS	Control	*Burkholderia fungorum*	99
15	1	SSMS	High	*Burkholderia fungorum*	99
24	1	SSMS	Med	*Burkholderia fungorum strain*	98
21	1	SSMS	High	*Cellulomonas cellasea*	99
11	1	Artificial	Med	*Crocinobacterium jejui*	99
41	1	SSMS	Low	*Herminiimonas arsenicoxydans*	96
7	1	SSMS	Med	*Hymenobacter ocellatus*	96
45	1	Artificial	Low	*Kaistia adipata*	99
16B	3	SSMS	High	*Knoellia sinensis*	99
53	1	SSMS	Low	*Microbacterium lacus*	99
16A	(3)	SSMS	Low	*Microbacterium lacus*	100
38	1	SSMS	Low	*Paenibacillus tundra*	100
56	1	SSMS	Med	*Paenibacillus wynnii*	99
13A	(15)	SSMS	High	*Pseudomonas brenneri*	99
13B	(15)	Artificial	High	*Pseudomonas putida*	99
13C	(15)	Artificial	High	*Pseudomonas putida*	99
13E	(15)	SSMS	High	*Pseudomonas migulae*	99
10	7	Artificial	Low	*Pseudomonas frederiksbergensis*	99
46	2	Artificial	Low	*Pseudomonas frederiksbergensis*	99
4A	(21)	Artificial	Control	*Pseudomonas thivervalensis*	98
4B	(21)	Artificial	Low	*Pseudomonas frederiksbergensis*	99
4C	(21)	SSMS	High	*Pseudomonas mandelii*	100
12	8	SSMS	High	*Pseudomonas migulae*	98
19	1	Artificial	High	*Pseudomonas thivervalensis*	98
30	1	SSMS	Low	*Pseudomonas veronii*	99
44	1	Artificial	Med	*Pseudoxanthomonas yeongjuensis*	99
22	1	SSMS	High	*Rahnella aquatilis*	98
14	2	SSMS	Low	*Rhodanobacter fulvus*	99
67	1	SSMS	High	*Rhodanobacter ginsengisoli*	98
68	2	SSMS	High	*Rhodanobacter spathiphylli*	98
9	1	Artificial	Med	*Rhodococcus baikonurensis*	100
18A	2	Artificial	Med	*Rhodococcus baikonurensis*	99
18B	(2)	Artificial	Med	*Rhodococcus baikonurensis*	100
58	1	SSMS	Med	*Rhodococcus erythropolis*	99
43	3	Artificial	Med	*Rhodococcus erythropolis*	99
2	(39)	Artificial	Med	*Rhodococcus globerulus*	99
40	1	SSMS	Low	*Sphingomonas echinoides*	99
37A	(3)	SSMS	Low	*Sphingomonas aquatilis*	99
37B	(3)	SSMS	Low	*Sphingomonas echinoides*	99
17B	(34)	SSMS	Low	*Sphingomonas echinoides*	100
17C	(34)	Artificial	High	*Sphingomonas faeni*	100
17D	(34)	Artificial	High	*Sphingomonas faeni*	99
17G	(34)	SSMS	High	*Sphingomonas faeni*	100
57	1	SSMS	High	*Sphingomonas faeni*	95
17F	(34)	SSMS	Low	*Sphingomonas rhizogenes*	99
6	3	Artificial	Low	*Sphingomonas oligophenolica*	99
49	1	SSMS	Low	*Sphingomonas oligophenolica*	99
17H	(34)	SSMS	Low	*Sphingomonas oligophenolica*	99
8	1	SSMS	High	*Sphingomonas oligophenolica*	99
5B	2	Artificial	High	*Sphingomonas oligophenolica*	100
62	8	SSMS	Control	*Sphingomonas sanxanigenens*	99
69	4	SSMS	Low	*Sphingomonas sanxanigenens*	99
28	1	SSMS	Low	*Sphingomonas sanxanigenens*	96
5A	3	SSMS	Low	*Sphingomonas sanxanigenens*	99
59	1	SSMS	Control	*Sphingopyxis flavimaris*	100
29	1	SSMS	Control	*Sporosarcina globispora*	99
48	1	Artificial	Low	*Sporosarcina globispora*	99
51	1	SSMS	Control	*Sporosarcina psychrophila*	99
23	1	SSMS	High	*Yersinia enterocolitica*	96

Where multiple identical RFLPs were recovered, 2–7 isolates were sequenced. The RFLP numbers presented in brackets indicate the total number of identical RFLPs recovered, irrespective of total number of isolates sequenced.

**Table 4:** The most significantly inhibited and stimulated genera present in gDNA recovered from the SSMS enrichments or directly from soil.

Genera^b^	Phyla^c^	Slope	P value	Relative abundance in control %	Relative abundance high TPH % (average) (SD)	
**SSMS Inhibited**^a^						
*Rhodoferax*	*β-proteo*.	−0.41	0.02	13.3	0.2	0.3
*Simplicispira*	*β-proteo*.	−0.35	0.02	15.0	1.1	1.6
unc. *Comamonadaceae*	*β-proteo*.	−0.24	0.01	3.8	Undetected	n.a
unc. *Rhodocyclaceae*	*β-proteo*.	−0.21	0.02	4.4	Undetected	n.a
unc. *Bacteroidetes*	*Bacteroid*.	−0.18	0.03	2.9	Undetected	n.a
*Methylophilus*	*β-proteo*.	−0.15	0.04	1.2	Undetected	n.a
*Malikia*	*β-proteo*.	−0.15	0.02	1.4	Undetected	n.a
*Lactobacillus*	*Firmicut*.	−0.14	0.01	8.8	2.7	0.9
*Flexithrix*	*Bacteroid*.	−0.11	0.03	1.3	Undetected	n.a
*Dechloromonas*	*β-proteo*.	−0.10	0.02	1.0	Undetected	n.a
**SOIL Inhibited**^a^						
*Opitutus*	*Verrucom*.	−0.24	0.02	4.5	0.5	0.6
unc. Bacteria	n.a.	−0.23	0.04	8.2	1.4	0.1
unc. *Xanthomonadaceae*	*γ-proteo*.	−0.21	0.01	3.6	0.5	0.3
unc. *γ-proteobacteria*	*γ-proteo*.	−0.20	0.02	4.7	0.5	0.0
*Steroidobacter*	*γ-proteo*.	−0.20	0.01	3.1	0.5	0.3
unc. *Chloroflexi*	*Chlorofl*.	−0.19	0.02	6.2	1.4	0.2
*Geminicoccus*	*α-proteo*.	−0.19	0.04	2.7	0.2	0.2
*Gemmatimonas*	*Gemmati*.	−0.19	0.02	7.4	2.6	0.7
unc. *β-proteobacteria*	*β-proteo*.	−0.18	0.04	4.3	0.8	0.3
unc. *Planctomycetaceae*	*Planctom*.	−0.18	0.02	2.7	0.3	0.1
**SSMS Stimulated**						
*Herbaspirillum*	*β-proteo*.	0.26	0.02	0.3	3.0	1.0
*Dyella*	*γ-proteo*.	0.23	0.04	0.7	2.3	2.5
*Janthinobacterium*	*β-proteo*.	0.20	0.01	0.7	3.7	0.4
*Parvibaculum*	*α-proteo*.	0.19	0.02	0.0	2.3	1.2
*Pseudomonas*	*γ-proteo*.	0.14	0.01	8.7	19.1	2.3
*Azotobacter*	*γ-proteo*.	0.05	0.05	0.0	0.3	0.1
**SOIL Stimulated**						
*Pseudomonas*	*γ-proteo*.	0.34	0.01	0.5	8.8	1.8
*Parvibaculum*	*α-proteo*.	0.27	0.00	0.7	3.4	3.3
*Herbaspirillum*	*β-proteo*.	0.15	0.03	0.1	1.1	1.3
*Dyella*	*γ-proteo*.	0.13	0.03	1.5	3.4	2.3
*Halotalea*	*γ-proteo*.	0.13	0.01	0.0	0.7	0.8

^a^Only the 10 most inhibited genera from each method were included in this list.

^b^If an OTU was unable to be classified to the genera level (unc.) and the closest classification was used.

^c^abbreviations for Phyla (*proteo*. = *proteobacteria*, *Firmicut*. = *Firmicutes*, *Bacteroid*. = *Bacteroidetes*, *Verrucom* = *Verrucomicrobia*, *Gemmati*. = *Gemmatimonadetes*, *Planctom*. = *Planctomycetes*).
